# Sibling Relationships of Adolescents with Congenital Heart Disease

**DOI:** 10.3390/ijerph18052698

**Published:** 2021-03-08

**Authors:** Youngji Moon, Jo Won Jung, Sunhee Lee

**Affiliations:** 1 College of Nursing, The Catholic University of Korea, Seoul 03722, Korea; myj2037@catholic.ac.kr; 2 Division of Pediatric Cardiology, Department of Pediatrics, College of Medicine, Yonsei University, Seoul 03722, Korea; JWJUNG@yuhs.ac

**Keywords:** sibling relationships, relative power, rivalry, congenital heart disease

## Abstract

Adolescents with congenital heart disease (CHD) continuously need family support because of their repeated follow ups, treatments, and complications. However, sibling relationships have not been well studied among adolescents with CHD. The purpose of the present study was to explore the relationships between adolescents with CHD and their siblings, and to examine these relationships according to birth order and age. Adolescents aged from 13 to 21 years who had been diagnosed with CHD and had siblings were included as participants. The Sibling Relationship Questionnaire (SRQ) was used. The SRQ consists of four factors: warmth/closeness, conflict, relative power/status, and rivalry. A univariate general linear model was conducted to identify the sibling relationship factors according to birth order and sibling ages. The score for relative power/status of participants who were the eldest sibling was higher than that of younger siblings. The score for rivalry increased as sibling age increased. Therefore, healthcare providers need to investigate sibling relationships and to explain the importance of self-identity and power balance between adolescents with CHD and their siblings to parents.

## 1. Introduction

Congenital heart disease (CHD) is now considered to be a chronic illness due to advanced improvements in treatments [1]. Children and adolescents with CHD need continuous family support because of their repeated follow ups, treatments, and complications [2]. Since CHD is now considered a chronic illness, many studies of the relationship between CHD children and their parents have been conducted [2,3]. However, the relationships between CHD adolescents and their siblings is under studied [4]. Sibling relationships last throughout the lifespan [5] and can greatly influence future interpersonal relations and identity development [6]. Additionally, it is an important factor affecting psychological functioning [7]. Parents of children with CHD tend to focus on the child with a disease and to feel sorry for their healthy children who are neglected [2]. In these situations, children felt they received differential treatment. Differential treatment refers to a children’s perception that their parents behaved differently towards them than towards their siblings [8].

Siblings of children with chronic illness might be in more difficult situations compared to those of healthy children. Dinleyici et al. [9] reported that the quality of life of siblings of children with chronic illness was lower than that of healthy children. Health and quality of life of siblings were affected by having a brother or sister with CHD [10]. Some studies reported that siblings of a child with chronic illness had caregiving roles and responsibilities [11,12]. Sometimes they felt they were the ‘forgotten’ member of the family [11] and had to assist their parents or to substitute for their parent’s role in the care of the child with a chronic illness [12]. In addition, they experienced negative emotions such as fear of the chronic illness and felt their family was not normal [13]. These situations can affect both siblings and CHD adolescents negatively because siblings affect each other’s emotional and social wellbeing [14].

During adolescence, growing children are faced with psychosocial fluctuation [14] and development of identity which are greatly influenced by their sibling relationships [6]. Especially, some researchers have reported that sibling relationships are affected by birth order and age [15,16]. Sibling conflict tended to decrease with age [16,17] and a low degree of behavioral problems were shown in case of older siblings with a younger children with CHD [10]. However, even descriptive studies that investigated the sibling relationships among CHD adolescents according to general characteristics are rare. Both CHD adolescents and their siblings may be in a difficult situation with respect to establishing appropriate sibling relationships. Therefore, the aim of the present study was to explore the sibling relationships of CHD adolescents and to examine these relationships according to birth order and age. The hypotheses of this study was that the adolescents with CHD (1) who are the first-born and (2) whose sibling are old would show a higher level of warmth and a lower level of conflict, relative power, and rivalry among the sibling relationships

## 2. Materials and Methods

### 2.1. Setting and Participants

Our target sample included adolescents 13 to 21 years old who were enrolled in the Pediatric Cardiology department of a university-affiliated tertiary medical center located in Seoul; had been diagnosed with CHD; had one or more treatments or surgeries related to the disease; had siblings, and who understood the purpose of the study, and voluntarily agreed to participate in the study.

Sample size was calculated as 92 using the G*power 3.1.9.4 (HHU, Dusseldorf, Germany) program with a significance level of 0.05, power of 80%, and an effect size of 0.15. Given the probability of participant attrition, at least 120 participants were required. An initial sample of 120 participants was recruited for the study. After excluding 11 people who did not meet the inclusion criteria, refused to participate in the study, or failed to complete the survey, data collected from 109 participants were used for analysis.

### 2.2. Data Collection Procedure

After obtaining approval from the Institutional Review Board (4-2019-0940), data were collected from 2 January to 28 February 2020. When adolescents with CHD visited the Pediatric Cardiology outpatient clinic, the researcher explained the purpose of the study, its benefits and risks, voluntary participation and withdrawal during the study, and confidentiality. Then, after acquiring consent from adolescents and their parents, the researcher collected data. The researcher explained how to complete a questionnaire and the self-reported questionnaire was given to adolescents to fill out in the waiting room of the outpatient clinic.

### 2.3. Measurement

#### Sibling Relationship Questionnaire (SRQ)

The sibling relationship perceived by adolescents with CHD was measured using the Sibling Relationship Questionnaire (SRQ) developed by Furman and Buhrmester [18]. The SRQ consists of 48 items and assesses four factors: warmth/closeness, conflict, relative power/status, and rivalry. Warmth/closeness consists of prosocial behavior, affection, companionship, similarity, intimacy, admiration of siblings, and admiration by sibling. Conflict consists of antagonism, quarreling, and competition. Relative power/status consists of sibling nurturance, sibling nurturance, sibling dominance, and dominance by a sibling. Rivalry consists of paternal and maternal partiality. A five-point Likert scale is used and higher scores indicate a higher value for each factor in the sibling relationship. If the participants had more than one sibling, they choose one sibling who influenced them most. The Korean version of the SRQ was verified for reliability and validity by Park [19] and was used in this study. According to the reliability of the instrument by Park, the Cronbach α value for warmth/closeness, conflict, relative power/status, and rivalry were 0.93, 0.85, 0.81, and 0.77, respectively [19]. In this study, the Cronbach α value for warmth/closeness, conflict, relative power/status, and rivalry were 0.96, 0.92, 0.73, and 0.65, respectively.

### 2.4. Data Analysis

Data were analyzed using the SPSS 21.0 statistics program (IBM Corp., Armonk, NY, USA). Descriptive statistics were used to identify the general characteristics and four factors of sibling relationships (warmth/closeness, conflict, relative power/status, and rivalry). Independent t-tests, analysis of variance (ANOVA), and the post hoc Schéffe test were performed for sibling relationships according to the general characteristics. A univariate general linear model was performed to identify the differences of sibling relationships according to birth order and sibling age at designating the variables which were significantly different in the previous analysis as covariates.

## 3. Results

### 3.1. General Characteristics of Adolescents with CHD, Parents, and Siblings

Of the 109 participants, 61 (55.5%) were male and 49 (44.5%) were female. Fifty-four (49.1%) were aged 13 to 15 and most participants attended middle school (40.0%) and high school (35.5%). Sixty-three (57.3%) received heart surgery once and the majority of participants did not have any complications (90.9%). As for the severity of CHD based on the classification by Warnes et al. [20] 15 adolescents (13.8%) were classified with simple severity, 61 (56.0%) were classified with moderate severity and 33 (30.3%) were classified as great complexity of CHD. The birth order for first sibling and being the second or later sibling was 40.9% and 59.1%, respectively. Regarding parent age, 61 (56%) of fathers and 87 (79.1%) of mothers were under the age of 49. As for siblings, 62 (56.4%) were male and 80 (72.7%) were under the age of 18. (Table 1).

### 3.2. Sibling Relationships with CHD Adolescents

The mean scores for warmth/closeness, conflict, relative power/status, and rivalry in sibling relationships were 2.90, 2.54, 3.03 and 3.16, respectively (Table 2).

### 3.3. Sibling Relationships According to Participant General Characteristic

As shown in Table 3, warmth/closeness among the sibling relationships factors was significantly related to age. Conflict was significantly related to sex, age, education level, and birth order. Relative power/status was significantly related to birth order and there was no variable that was significantly related to rivalry.

### 3.4. Sibling Relationships According to General Characteristics of the Parents and Siblings

As shown in Table 4, warmth/closeness was significantly related to the father’s age and father’s educational level. Conflict was significantly related to the father’s age, mother’s age, sibling’s sex, and sibling’s age. Father’s age, mother’s age, and sibling’s age showed statistically significant differences related to sibling relationship power/status. Father’s education level and sibling’s age showed statistically significant differences related to rivalry.

### 3.5. Sibling Relationship According to Birth Order and Sibling’s Age

**Figure 1:**
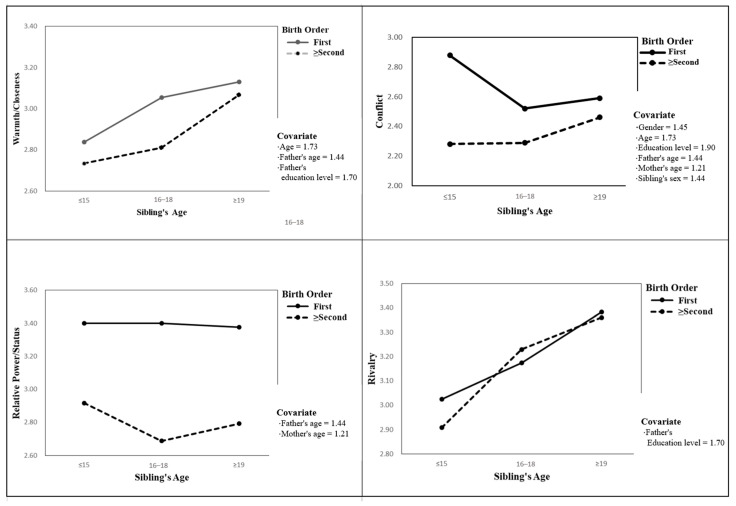
Factors of sibling relationships according to birth order and sibling’s age.

Figure 1 presents the four factors (warmth/closeness, conflict, relative power/status, and rivalry) of sibling relationships according to birth order and sibling’s age. The score for relative power/status of the participants who were the eldest was higher than younger ages. The score for rivalry increased as sibling age increased.

## 4. Discussion

The means for relative power/status and rivalry scores in this study, which are the factors of sibling relationships, were 3.03 and 3.16. The results of the present study indicate that the mean scores of relative power/status and rivalry were high compared to those of other similar studies in Western countries [21,22]. To be specific, the mean scores for relative power/status and rivalry between 10–13 years old adolescents in Canada were 2.52 and 1.89, and those between siblings over 18 years of age in New Zealand were 2.5 and 2.95. The mean scores for relative power/status and rivalry of 10–17 years old adolescents with a sibling having developmental disability in the US were 2.14 and 2.93 [23] and those for 8–12 years old children with a sibling having ADHD in Sweden were 2.45 and 2.92 [24].

With regard to relative power/status, the results of this study indicate that the scores for relative power/status did not change with advancing age and relative power/status was different by birth order. The power/status of the eldest child was distinctly higher than younger children, regardless the participants in this study have had the CHD. Since Korean families are still affected by egalitarian family norms as well as Confucian family norms [25], the ideal of ‘eldest come first’ still tends to influence on Korean families including the adolescents with CHD. Inconsistent with this study, sibling relationships tend to change from hierarchical in early childhood to horizontal by adulthood and the relative power/status imbalance is significantly decreased over the course of adolescence [16]. However, relative power/status of the adolescents in this study did not changed from hierarchical to horizontal. Begum and Blacher [26] reported that the relative power/status among sibling relationships can affect behavioral problems including internalizing and externalizing problems. Sibling posturing for power likely occurs during conflict [16], and power imbalance between siblings was associated with aggressive and coercive behavior and was linked to internalizing and externalizing behavior problems [15]. Therefore, further studies on relative power/status of the adolescents with CHD and their siblings are needed and healthcare providers need to explain the importance of power balance between adolescents with CHD and their siblings to parents.

The results of this study indicate that rivalry was increased as siblings advanced in years of age and. Sibling rivalry refers to feelings of envy, jealously, and competitiveness that exist between brothers and sisters within the family [17]. Inconsistent with this study, Volling et al. [17] reported that sibling rivalry declines as adolescents grow in older. Sulloway [27] mentioned that sibling difference is from disparities in parental investment and siblings tend to develop personal niches within the family so as to encourage an optimal level of parental investment as well as to reduce competition with other siblings. Siblings often seek to differentiate themselves within the family [27] and differentiation between self and others allows autonomy and identity from a realistic appraisal of self and others (Goth &, 2012). The difference between siblings can be considered meaningful. Difference fosters closeness among siblings and differentiation eases the tensions of sibling rivalry [28]. However, since Korean culture has been framed by Confucianism, children are forced to do some of best things as a child’s role such as studying hard to enter the prestigious university [29]. Korean children tend to do the same thing for their future without any opportunities to find their own characteristics or interests. Sibling rivalry was positively correlated with hostility among siblings [17] and it can lead to competitive personalities, lasting personal difficulties [6], and can be an obstacle to establishing self-identity [28]. However, sibling relationships are ambivalent, love and hate, and difficult and envious relationships between siblings exist in everyday life [30]. Therefore, Differentiation used to resolve conflicts with siblings and facilitate specialization of role. Additionally, healthcare providers need to support adolescents with CHD and their sibling to have differentiation and identity.

### Limitations

Sibling relationships can be grasped accurately by gathering opinions from both adolescents with CHD and their siblings. However, the results of the present study are reported from the sibling relationships scores of adolescents with CHD. Future research that investigates sibling relationships from both sides is needed. Sibling relationships can be affected by the dyad which was the same sex or the opposite [26]. However, the present study could not consider sex of dyad, therefore, future study for the sibling relationships considering the sex of dyad is needed. Since the age range of the present study was wide, future research using age range based on the criteria of developmental psychology is needed. Additionally, the present study could not consider the drug that the participants took and the half-sibling, thus future research need to take into account the half-sibling and taken drugs that the present study did not consider.

## 5. Conclusions

The results of the present study indicate that the mean scores for rivalry and relative power/status were high compared to those of other studies in Western countries, and the relative power/status of CHD adolescents was different by birth order. These power imbalances between the eldest child and others can increase behavioral problems in CHD adolescents and their siblings. In addition, sibling rivalry for adolescents with CHD increased as sibling age increased. Sibling rivalry can be an obstacle to establishing self-identity and negatively influences CHD adolescents and their siblings. Therefore, healthcare providers need to investigate sibling relationships, especially relative power/status and rivalry, for adolescents with CHD and their siblings.

## Figures and Tables

**Table 1:** Demographic characteristics of participants.

Variable	Categories	*n*	%
**Characteristics of participants**			
Sex	Male	61	55.5
	Female	49	44.5
Age	13–15	54	49.1
	16–18	32	29.1
	19–21	24	21.8
Education level	Middle school	44	40.0
	High school	39	35.5
	College	21	19.1
	Others	6	5.5
Frequency of heart surgery	1	63	57.3
	2	26	23.6
	3	12	10.9
	4	9	8.2
Complications	Yes	10	9.1
	No	100	90.9
Medicine	Yes	61	55.5
	No	49	44.5
NYHA class	1	65	59.1
	2	40	36.4
	3	5	4.5
Severity of CHD	Simple	15	13.8
	Moderate severity	61	56.0
	Great complexity	33	30.3
Birth order	First	45	40.9
	≥Second	65	59.1
**Characteristics of parents and siblings**			
Father’s age	≤49	61	56.0
	≥50	48	44.0
Mother’s age	≤49	87	79.1
	≥50	23	20.9
Father’s education level	≤High school	45	41.3
	College	53	48.6
	Graduate school or beyond	11	10.1
Mother’s education level	≤High school	50	45.9
	College	53	48.6
	Graduate school or beyond	6	5.5
Sibling’s sex	Male	62	56.4
	Female	48	43.6
Sibling’s age	≤15	46	41.8
	16–18	34	30.9
	≥19	30	27.3

**Table 2:** The levels of sibling relationships.

Variable	Sub-Categories	Mean ± SD	Item Number	Range
Sibling relationships	Warmth/Closeness	2.90 ± 0.84	21	1~5
	Conflict	2.54 ± 0.96	9	
	Relative Power/Status	3.03 ± 0.57	12	
	Rivalry	3.16 ± 0.55	6	
	Total	2.90 ± 0.44	48	

**Table 3:** Sibling relationships according to participant characteristics.

Variables	Warmth/Closeness		Conflict		Relative Power/Status		Rivalry	
	Mean ± SD	t or F (*p*)	Mean ± SD	t or F (*p*)	Mean ± SD	t or F (*p*)	Mean ± SD	t or F (*p*)
Sex								
Male	2.96 ± 0.75	0.75 (0.46)	2.26 ± 0.86	−3.69 (0.00)	2.94 ± 0.54	−1.91 (0.06)	3.17 ± 0.64	0.13 (0.89)
Female	2.83 ± 0.94		2.90 ± 0.97		3.14 ± 0.58		3.16 ± 0.42	
Age								
13–15	2.70 ± 0.87	3.15 (0.05)	2.88 ± 1.01	7.52 (0.00)	3.04 ± 0.64	0.04 (0.96)	3.18 ± 0.52	0.30 (0.74)
16–18	3.05 ± 0.72		2.26 ± 0.89		3.04 ± 0.49		3.10 ± 0.53	
19–21	3.14 ± 0.84		2.15 ± 0.64		3.00 ± 0.50		3.22 ± 0.66	
Education level								
Middle school	2.74 ± 0.88	1.6 (0.19)	2.93 ± 1.02	4.29 (0.01)	3.09 ± 0.63	0.63 (0.60)	3.18 ± 0.53	0.94 (0.43)
High school	2.94 ± 0.79		2.30 ± 0.95		2.99 ± 0.55		3.15 ± 0.51	
College	3.21 ± 0.80		2.24 ± 0.67		2.94 ± 0.51		3.25 ± 0.66	
Others	2.79 ± 0.91		2.39 ± 0.50		3.21 ± 0.24		2.83 ± 0.59	
Frequency of heart surgery								
1	2.85 ± 0.91	0.24 (0.87)	2.63 ± 0.93	1.50 (0.22)	3.00 ± 0.53	2.44 (0.07)	3.19 ± 0.58	0.36 (0.79)
2	3.00 ± 0.73		2.63 ± 1.04		3.26 ± 0.69		3.19 ± 0.55	
3	2.98 ± 0.77		2.30 ± 0.76		2.81 ± 0.34		3.01 ± 0.43	
4	2.84 ± 0.77		2.00 ± 1.07		2.86 ± 0.48		3.13 ± 0.57	
Complications								
Yes	3.03 ± 0.72	0.52 (0.60)	2.28 ± 1.12	−0.92 (0.36)	2.85 ± 0.47	−1.05 (0.29)	3.35 ± 0.70	1.12 (0.26)
No	2.89 ± 0.85		2.57 ± 0.94		3.05 ± 0.57		3.15 ± 0.53	
Medicine								
Yes	2.89 ± 0.85	0.15 (0.89)	2.43 ± 1.05	1.39 (0.17)	2.98 ± 0.56	1.12 (0.26)	3.12 ± 0.50	0.98 (0.33)
No	2.91 ± 0.84		2.68 ± 0.83		3.10 ± 0.58		3.22 ± 0.61	
NYHA class								
Ⅰ	2.92 ± 0.85	0.15 (0.86)	2.52 ± 0.90	0.26 (0.77)	3.07 ± 0.51	0.44 (0.65)	3.18 ± 0.51	0.33 (0.72)
Ⅱ	2.85 ± 0.88		2.54 ± 0.99		2.96 ± 0.66		3.17 ± 0.62	
Ⅲ	3.03 ± 0.41		2.84 ± 1.52		3.05 ± 0.60		2.97 ± 0.61	
Severity of CHD								
Simple	3.08 ± 1.01	0.86 (0.43)	2.47 ± 0.99	1.33 (0.27)	3.14 ± 0.44	1.33 (0.27)	3.22 ± 0.62	0.19 (0.83)
Moderate severity	2.94 ± 0.82		2.68 ± 0.94		3.08 ± 0.60		3.17 ± 0.56	
Great complexity	2.76 ± 0.82		2.34 ± 0.99		2.90 ± 0.54		3.12 ± 0.53	
Birth order								
First	2.85 ± 0.86	−0.57 (0.57)	2.81 ± 0.93	2.45 (0.02)	3.44 ± 0.39	8.07 (0.00)	3.06 ± 0.48	−1.73 (0.09)
≥Second	2.94 ± 0.83		2.36 ± 0.95		2.74 ± 0.48		3.24 ± 0.59	

**Table 4:** Sibling relationships according to characteristics of parents and siblings.

Variables	Warmth/Closeness		Conflict		Relative Power/Status		Rivalry	
	Mean ± SD	t or F (p)	Mean ± SD	t or F (p)	Mean ± SD	t or F (p)	Mean ± SD	t or F (p)
Father’s age								
≤49	2.71 ± 0.83	−2.66 (0.01)	2.70 ± 1.02	2.01 (0.05)	3.22 ± 0.55	4.67 (0.00)	3.10 ± 0.46	−1.41 (0.16)
≥50	3.13 ± 0.79		2.34 ± 0.85		2.76 ± 0.46		3.25 ± 0.65	
Mother’s age								
≤49	2.86 ± 0.87	−0.93 (0.36)	2.71 ± 0.96	3.65 (0.00)	3.11 ± 0.57	3.05 (0.00)	3.15 ± 0.52	−0.59 (0.55)
≥50	3.05 ± 0.73		1.93 ± 0.69		2.72 ± 0.43		3.22 ± 0.67	
Father’s education level								
≤High school	2.70 ± 0.76	3.43 (0.04)	2.61 ± 0.91	0.56 (0.57)	2.99 ± 0.54	1.01 (0.37)	3.02 ± 0.40	5.35 (0.01)
College	3.12 ± 0.91		2.53 ± 0.93		3.10 ± 0.57		3.20 ± 0.57	
Graduate school or beyond	2.78 ± 0.47		2.26 ± 1.32		2.86 ± 0.66		3.59 ± 0.79	
Mother’s education level								
≤High school	2.85 ± 0.74	1.89 (0.16)	2.68 ± 0.97	2.23 (0.11)	2.98 ± 0.52	2.09 (0.13)	3.13 ± 0.54	0.48 (0.62)
College	3.03 ± 0.91		2.48 ± 0.90		3.12 ± 0.62		3.18 ± 0.57	
Graduate school or beyond	2.38 ± 0.70		1.85 ± 1.21		2.67 ± 0.35		3.36 ± 0.59	
Sibling’s sex								
Male	2.94 ± 0.83	0.56 (0.58)	2.37 ± 0.94	−2.23 (0.03)	3.00 ± 0.61	−0.56 (0.57)	3.18 ± 0.61	0.36 (0.72)
Female	2.85 ± 0.85		2.77 ± 0.95		3.06 ± 0.50		3.14 ± 0.46	
Sibling’s age								
≤15	2.74 ± 0.85	2.89 (0.06)	2.90 ± 0.96	6.21 (0.00)	3.34 ± 0.51	16.43(0.00)	2.98 ± 0.45	5.07 (0.01)
16–18	2.86 ± 0.82		2.38 ± 0.93		2.89 ± 0.53		3.24 ± 0.50	
≥19	3.20 ± 0.79		2.19 ± 0.82		2.71 ± 0.45		3.36 ± 0.66	

## Data Availability

The data presented in the present study are available on request from the corresponding author.
